# Evaluation of Autonomic Nervous System Function During Sleep by Mindful Breathing Using a Tablet Device: Randomized Controlled Trial

**DOI:** 10.2196/56616

**Published:** 2024-06-12

**Authors:** Eiichi Togo, Miki Takami, Kyoko Ishigaki

**Affiliations:** 1 Department of Nursing, Faculty of Nursing, Hyogo University Kakogawa City Japan; 2 College of Nursing Art and Science, University of Hyogo Akasi City Japan

**Keywords:** mindfulness, sleep, cardiac potential, low frequency, high frequency, mobile phone

## Abstract

**Background:**

One issue to be considered in universities is the need for interventions to improve sleep quality and educational systems for university students. However, sleep problems remain unresolved. As a clinical practice technique, a mindfulness-based stress reduction method can help students develop mindfulness skills to cope with stress, self-healing skills, and sleep.

**Objective:**

We aim to verify the effectiveness of mindful breathing exercises using a tablet device.

**Methods:**

In total, 18 nursing students, aged 18-22 years, were randomly assigned and divided equally into mindfulness (Mi) and nonmindfulness (nMi) implementation groups using tablet devices. During the 9-day experimental period, cardiac potentials were measured on days 1, 5, and 9. In each sleep stage (sleep with sympathetic nerve dominance, shallow sleep with parasympathetic nerve dominance, and deep sleep with parasympathetic nerve dominance), low frequency (LF) value, high frequency (HF) value, and LF/HF ratios obtained from the cardiac potentials were evaluated.

**Results:**

On day 5, a significant correlation was observed between sleep duration and each sleep stage in both groups. In comparison to each experimental day, the LF and LF/HF ratios of the Mi group were significantly higher on day 1 than on days 5 and 10. LF and HF values in the nMi group were significantly higher on day 1 than on day 5.

**Conclusions:**

The correlation between sleep duration and each sleep stage on day 5 suggested that sleep homeostasis in both groups was activated on day 5, resulting in similar changes in sleep stages. During the experimental period, the cardiac potentials in the nMi group showed a wide range of fluctuations, whereas the LF values and LF/HF ratio in the Mi group showed a decreasing trend over time. This finding suggests that implementing mindful breathing exercises using a tablet device may suppress sympathetic activity during sleep.

**Trial Registration:**

UMIN-CTR Clinical Trials Registry UMIN000054639; https://tinyurl.com/mu2vdrks

## Introduction

Recently, the spread of the internet and smartphones and the influence of companies operating around the clock have led to the rapid development of a society where people can be active at any time of the day or night. According to a survey by the Ministry of Health, Labor and Welfare, 37.5% (1000/2668) of men and 40.6% (1231/3033) of women sleep <6 hours, and 37.1% (82/221) of men and 37% (83/224) of women are in their 20s [1]. Approximately 37% (165/445) of university students in their 20s do not sleep sufficiently.

A study that investigated the relationship between sleep disturbances in university students and depression after graduation reported that those who developed sleep disturbances during their school years were at a higher risk of developing depression later in life [2]. Another study that investigated the association between sleep duration and impaired glucose tolerance found that sleeping for <6 hours was associated with an increased prevalence of diabetes [3]. Sleep disorders increase the risk of developing psychiatric disorders, cardiovascular diseases, and other physical diseases [4,5]. Therefore, as an issue to be considered in universities, improving the educational system for university students and providing interventions to improve sleep quality are necessary. The Ministry of Health, Labor and Welfare has established sleep guidelines for health promotion [6], and sleep-related consultations and support systems have been established at universities to help resolve sleep-related issues. VR use in adolescents with insomnia produces significant changes in heart rate, suggesting a relaxation effect [7]. Breathing interventions suggest that spontaneous slow breathing affects the parasympathetic nervous system [8], and breathing techniques may improve sleep quality and morning and evening cardiac vagal activity [9]. However, the impact of breathing on heart rate needs to be investigated via polysomnography to assess its effect on sleep, and its long-term efficacy in improving cardiovascular function is unknown [7,9]. Given these factors, sleep problems remain unresolved.

The mindfulness-based stress reduction (MBSR) method, developed by Kabat, has attracted attention [10]. MBSR involves developing mindfulness skills to help people cope with stress, improve self-healing skills, and sleep better. Studies on the effects of mindfulness on sleep have used subjective sleep rating scales [11,12] and cortisol levels during morning awakening during mindfulness practice [13]. The relationship between the practice of MBSR and the functioning of the autonomic nervous system during sleep remains poorly verified via physiological assessments. Autonomic function measurement using heart rate variability (HRV) analysis has been used to objectively assess sleep [14,15]. Mindfulness-based psychological interventions can reduce depressive symptoms [16].

The effects of mindfulness-based cognitive therapy in older adults with sleep disorders have been demonstrated objectively using polysomnography [17]. An unresolved issue is that studies using objective sleep measures, such as polysomnography, suggest that the effects of mindfulness on insomnia and sleep disorders are small compared with subjective reports [18]. Therefore, whether mindfulness-based interventions change sleep patterns or alter subjective sleep assessments remains unclear.

HRV analysis is effective in assessing sleep quality [19]. HRV analysis can enable an objective understanding of changes in the autonomic nervous system during sleep. Furthermore, wearable devices can assess pathological sleep conditions such as insomnia, sleep apnea, and hypertension [20]. Wearable devices can also assess sleep stages and disorders [21]. Based on the results of these studies, wearable devices have significant potential for use in sleep research and clinical practice. Furthermore, using wearable devices capable of measuring HRV during sleep is a viable method with sufficient validity for measuring the variability in sleep stages [22-24]. These studies have suggested that sleep assessment using wearable devices capable of measuring HRV is feasible.

Here, we hypothesized that participants who practiced mindful breathing techniques using a tablet device would show characteristics of autonomic nervous system function during sleep. This study aimed to verify the effects of mindfulness breathing exercises using a tablet device and capture changes in autonomic nervous system function.

## Methods

### Recruitment

In a study, 27 participants were evaluated for HRV during stable sleep [25]. This study was conducted with a sample size of >20 participants. In contrast, 15 participants were evaluated for sleep quality using HRV [26]. Further, 20 participants were evaluated for sleep using electroencephalography power spectral density [27]. The number of participants in this study was as follows: all previous studies had small sample sizes with <20 participants.

Therefore, based on previous studies, we hypothesized that differences between groups could be identified if we collected data from 18 participants.

With an expected dropout rate of 10%, the experiment was continued by collecting data from 10 participants in each group until data from 18 participants were available for analysis. In this study, the participants were 18 female nursing students at University A from the 1st to 4th year, aged 18-22 years. The participants were divided into 2 groups: a group that implemented (the Mi [mindfulness group]) and a group that did not implement mindfulness breathing exercises (the nMi [nonmindfulness group]). Each group consisted of 9 participants, and randomization was used to minimize the influence of bias in the number of participants and age differences between participants. To classify the participants into 2 groups, random numbers were assigned using the RAND function in the spreadsheet software Excel (Microsoft Corp). Participants were excluded from this study if they had previous meditation and mindfulness experience or had taken sleep-inducing drugs or other medications to assess the effects of mindful breathing techniques without error.

Exclusion criteria for health status were history of sleep disorders, use of sleep-inducing drugs, and inconsistent sleep-wake rhythms. Lifestyle exclusion criteria included those who worked part-time during nighttime hours and consumed alcohol or excess caffeine. Participants who met these conditions were excluded because they may have affected the effectiveness of the mindfulness breathing techniques.

### Experimental Structure

Between May 2022 and November 2023, each student participated in this experiment.

**Figure 1:**
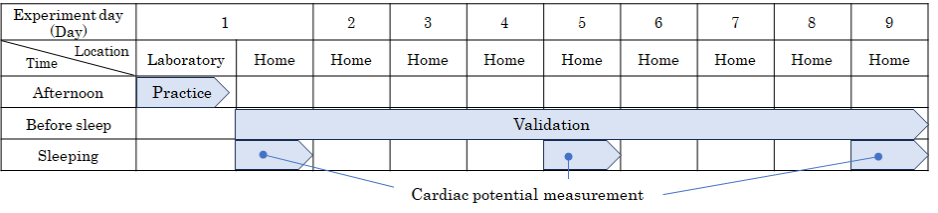
Experimental configuration. Practice: Mi group practicing mindful breathing exercises; nMi group practicing gazing at crosses. Validation: Mi group practicing mindful breathing exercises; nMi group practicing gazing at crosses.

On day 1 of the experiment, participants in the Mi group used the equipment and practiced mindful breathing exercises using a tablet device in a university laboratory. The nMi and control groups operated the equipment and practiced cross-gazing using a tablet device in a university laboratory. The laboratory practice was conducted in the afternoon to avoid the influence of the circadian cycle. The experimental structure consisted of 2 components: 1 “measurement” of cardiac potentials at home and the other “validation” of mindful breathing exercises or gazing at a cross on a personal computer screen (Figure 1). For the “measurement,” autonomic function was measured during sleep on days 1, 5, and 9 for the Mi and nMi groups. For the “validation,” the day 1 of the experiment was day 1, and for 9 consecutive weekdays, once a day at home before bedtime, the Mi group performed mindful breathing exercises, and the nMi group gazed at the crosshairs on the personal computer screen.

### Mindfulness

Mindfulness is described as “living consciously in the ‘now’ moment,” being aware of the present and accepting experience as it is, without evaluation or judgment [10]. Mindfulness aims to maintain moment-to-moment awareness and detach oneself from strong attachments to beliefs, thoughts, and feelings [28].

In this study, based on the MBSR developed by Kabat, an original Access (Microsoft Corp) application for voice guidance of mindful breathing exercises was created and experimented with. On day 1 of the experiment, the participants were provided a verbal overview of mindfulness in the laboratory and practiced mindful breathing exercises following audio guidance. The mindfulness procedure in this study consisted of (1) relaxing the shoulders and assuming a sitting posture (meditation); (2) maintaining the sitting posture, breathing abdominally, and focusing on the flow of exhalation and inhalation (breathing exercises); and (3) focusing attention sequentially on the fingertips, back, belly, chest, neck, and head while remaining aware of the breath and feeling the sensations in those areas (body scan) [10].

On day 9 of the experiment, at the end, a questionnaire was administered regarding the effects of mindful breathing exercises and participants’ intention to continue them. After explaining that the responses would be made confidential and statistically processed, the participants were asked to complete the questionnaire. The questionnaire included the following items regarding the effectiveness of mindful breathing exercises: “During the study, did implementing mindful breathing exercises have a positive effect on your sleep?” and asked them to respond on a 3-point scale (yes, no, or undecided). If the respondent answered “yes” to this question, she was asked, “What specific effects did it have?” and asked to describe the specific effects in free form.

### Cardiac Potential Measurements

Measuring the power spectrum values of HRV has been used as a noninvasive quantitative assessment during sleep [29]. Therefore, in this study, a wearable biometric sensor device was used to noninvasively measure cardiac potential and pulse waves from heartbeats in an environment similar to daily life. For the cardiac potential measurement, the Silmee Bar type Lite, an affixed wearable biometric sensor manufactured by TDK Corporation, which can noninvasively assess autonomic nervous system function, was used. The wearable biometric sensor used in this study could obtain data without disturbing the participants’ sleep [30]. For cardiac potential measurements, R-R intervals (RRI) were recorded at a sampling frequency of 1000 Hz. To remove body movement artifacts from the measurement data, the body movement error detection threshold of the RRI was set at 0.5 G. Measurements were made with a wearable biometric sensor attached approximately 3 cm below the middle of both clavicles to measure cardiac potentials close to the heart and reduce the influence of upper arm and chest muscle movement.

### Sleep Analysis

**Figure 2:**
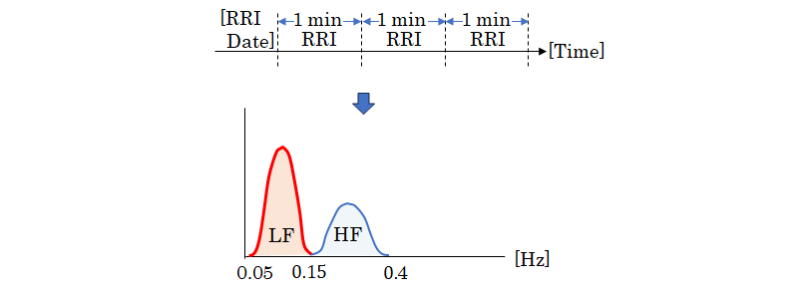
Overview of LF and HF calculations from RRI data. HF: high frequency; LF: low frequency; RRI: R-R interval.

In this study, Fast Fourier Transform was performed on the RRI of cardiac potential data during sleep using the Small_System manufactured by TDK Corporation. The low frequency (LF) components were separated into 0.05-0.15 and 0.15-0.40 Hz, with 1-min intervals, and the high frequency (HF) components were determined. The LF/HF ratio was calculated (Figure 2). The LF, HF, and LF/HF ratios are indices of HRV analysis and are used to assess autonomic function noninvasively. The LF component of HRV heart rate is jointly mediated by the sympathetic and parasympathetic nervous systems, whereas the HF component reflects only the parasympathetic nervous system, and the power ratio (LF/HF) reflects sympathetic activity [31,32]. The square root of the integral of the LF and HF power spectrum densities was calculated to suppress the variability and improve the accuracy of sleep determination. The time of sleep onset was estimated using Cole et al’s [33] method using acceleration data to determine the time of sleep onset, and the sleep stage classification was estimated based on the autonomic balance during the period when sleep was determined via acceleration data [34]. In this study, the acceleration sampling frequency of the accelerometer of the measurement device Small_System was recorded and analyzed at 125 Hz.

Sleep stages were assessed based on the relationship between autonomic activity and sleep stage and were divided into the following three stages: (1) sleep with sympathetic nerve dominance (S sleep), (2) shallow sleep with parasympathetic nerve dominance (PS sleep [shallow]), and (3) deep sleep with parasympathetic nerve dominance (PS sleep [deep]) [34]. As the sleep stages used in this study were measured using nonmedical equipment, expressions such as S sleep and PS sleep were used.

### Statistical Analysis

Statistical analyses were performed for the total sleep time, sleep stage (S sleep, PS sleep [shallow], and PS sleep [deep]), LF value, HF value, and LF/HF ratios. The normality of the total sleep time, sleep stage, LF value, HF value, and LF/HF ratio was tested using the Shapiro-Wilk test and Q-Q plots. In this study, the mean (SD) of total sleep time, each sleep stage, LF value, HF value, and LF/HF ratio on days 1, 5, and 9 of both groups were used for quantitative evaluation. *t* Tests (2-tailed) were performed on the Mi and nMi groups for total sleep time and sleep time per sleep stage on each experimental day to compare the groups. Correlation coefficients were used to evaluate effect sizes. Multiple comparisons of total sleep time and sleep time for each sleep stage within each group were performed using 1-way ANOVA and the Games-Howell method.

To test the effect of mindful breathing techniques, group comparisons were made for LF values, HF values, and the LF/HF ratio on days 1, 5, and 9 in both groups using *t* tests. Further, 1-way ANOVA was also performed for LF values, HF values, and LF/HF ratios between conditions on days 1, 5, and 9 in each group, and multiple comparisons were performed using the Games-Howell method. The results were rechecked using the Bonferroni correction. Statistical significance was set at 0.05. SPSS Statistics (version 26; IBM Corp) was used for statistical analysis.

### Ethical Considerations

All participants were fully informed of the purpose and objectives of this study, protection of their privacy, anticipated risks, the right to withdraw during this study, no disadvantages associated with this, publication of the results, guarantee of anonymity, and voluntary participation, and that the results would not be used for purposes other than research before this study commenced, and informed consent was obtained. The experiment was conducted in a private room, and efforts were made to protect the privacy of the participants. This study was approved by the Ethical Review Committee of the University of Hyogo and was conducted between May 2022 and December 2023 (21007) (Multimedia Appendix 1).

## Results

### Sleep Assessment Using a Questionnaire After Mindfulness Practice

Questionnaire test results were obtained from 6 of the 9 participants who practiced mindful breathing exercises using a tablet device. The valid response rate was 66.7% (6/9). All respondents answered “yes” to whether mindfulness meditation affected sleep quality. In free text responses, 1 respondent stated that she woke up feeling refreshed; 1 said that she felt calmer, which made it easier to sleep; 1 said that she was able to relax, get under the cover, sleep better, and was less likely to wake up in the middle of her sleep; and 2 said that they were able to fall asleep more easily.

### Sleep Duration

In this study, the total sleep time and stage (S sleep, PS sleep [shallow], and PS sleep [deep] times) for the Mi and nMi groups were tested for normality using the Shapiro-Wilk test and Q-Q plot, and they followed a normal distribution.

Table 1 shows the mean and SD of total sleep time and stages (S sleep, PS sleep [shallow], and PS sleep [deep]) for both groups on each experimental day and the *t* test results for total sleep time and stages for both groups. The *t* test results were not significant.

Table 2 shows the results of multiple comparisons of the total and mean sleep times for each sleep stage for the Mi and nMi groups on each experimental day. On each experimental day, significant differences were found in the mean values of total sleep time and sleep time for each sleep stage in both groups and in comparing the mean values of sleep time between the sleep stages. On each experimental day, no significant differences were found between S and PS sleep (deep) in either group.

**Table 1:** Total sleep time and stage time on the measurement day (N=18).

		Mi^a^, mean (SD)	nMi^b^, mean (SD)	*t* Test			Pearson correlation coefficient	
				*P* value		Significance	*r*	*P* value
**Day 1**								
	Total^c^	306 (107)	319 (109)	.80	ns^d^		0.369	.33
	S sleep^e^	58 (39)	77 (26)	.25	ns		–0.001	>.99
	PS (shallow)^f^	161 (83)	161 (64)	>.99	ns		0.346	.36
	PS (deep)^g^	68 (43)	59 (30)	.61	ns		–0.174	.65
**Day 5**								
	Total	381 (78)	332 (122)	.33^.^	ns		0.387	.30
	S sleep	81 (31)	48 (37)	.06	ns		–0.228	.56
	PS (shallow)	203 (64)	180 (79)	.52	ns		0.422	.26
	PS (deep)	67 (41)	56 (34)	.56	ns		–0.36	.34
**Day 9**								
	Total	342 (111)	336 (108)	.92	ns		0.793	.01
	S sleep	53 (37)	46 (32)	.67	ns		0.59	.09
	PS (shallow)	177 (69)	141 (59)	.25	ns		–0.029	.94
	PS (deep)	80 (26)	62 (24)	.14	ns		–0.245	.52

^a^Mi: mindfulness group.

^b^nMi: nonmindfulness group.

^c^Total: total sleep time.

^d^ns: not significant.

^e^S sleep: sleep with sympathetic nerve dominance.

^f^PS (shallow): shallow sleep with parasympathetic nerve dominance.

^g^PS (deep): deep sleep with parasympathetic nerve dominance.

**Table 2:** Association between total sleep time and stage using measurement date (N=18).

			*P* value		
			Mi^a^		nMi^b^
**Day 1**					
	**Total^c^**				
		S sleep^d^	3×10^–4^^e^	5×10^–4^^e^	
		PS (shallow)^f^	.027^g^	.01^g^	
		PS (deep)^h^	4×10^–4^^e^	2×10^–4^^e^	
	**S sleep**				
		PS (shallow)	.028^g^	.017^g^	
		PS (deep)	.959 ns^i^	.55 ns	
	**PS (shallow)**				
		PS (deep)	.05 ns	.005 ns	
**Day 5**					
	**Total**				
		S sleep	3×10^–6^^e^	3×10^–4^^e^	
		PS (shallow)	4×10^–4^^e^	.03^g^	
		PS (deep)	8×10^–7^^e^	4×10^–4^^e^	
	**S sleep**				
		PS (shallow)	.001^e^	.004^e^	
		PS (deep)	.855 ns	.965 ns	
	**PS (shallow)**				
		PS (deep)	5×10^–4^^e^	.006^e^	
**Day 9**					
	**Total**				
		S sleep	1×10^–4^^e^	1×10^–4^^e^	
		PS (shallow)	.01^g^	.002^e^	
		PS (deep)	3×10^–4^^e^	2×10^–4^^e^	
	**S sleep**				
		PS (shallow)	.002^e^	.005^e^	
		PS (deep)	.29 ns	.62 ns	
	**PS (shallow)**				
		PS (deep)	.01^g^	.017^g^	

^a^Mi: mindfulness group.

^b^nMi: nonmindfulness group.

^c^Total: total sleep time.

^d^S sleep: sleep with sympathetic nerve dominance.

^e^*P*<.01.

^f^PS (shallow): shallow sleep with parasympathetic nerve dominance.

^g^*P*<.05.

^h^PS (deep): deep sleep with parasympathetic nerve dominance.

^i^ns: not significant.

### Cardiac Potential Measurement

In this study, the LH values, HF values, and LF/HF ratios of the Mi and nMi groups were tested for normality using the Shapiro-Wilk test and Q-Q plots, and they followed a normal distribution.

**Figure 3:**
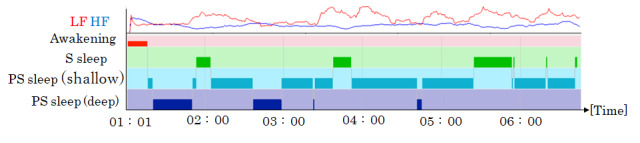
Example of the autonomic balance in LF and HF values and the chronological changes in each sleep stage. HF: high frequency; LF: low frequency; PS sleep (deep): deep sleep with parasympathetic nerve dominance; PS sleep (shallow): shallow sleep with parasympathetic nerve dominance; S sleep: sleep with sympathetic nerve dominance.

Figure 3 shows an example of the time course of autonomic balance in LF and HF values and sleep time in each sleep stage, where PS sleep (deep) was observed immediately after sleep onset.

Table 3 shows the results of the 1-way ANOVA comparing the LH and HF values and the LF/HF ratio for each experimental day and Bonferroni correction. The threshold for the Bonferroni correction was set at α/k, where α is the significance level, and k is the number of comparisons. When multiple comparisons and 1-way ANOVA were conducted, and the threshold for Bonferroni correction was set to α/3, the results using multiple comparisons and Bonferroni correction were also significantly different, consistent with the original results.

**Figure 4:**
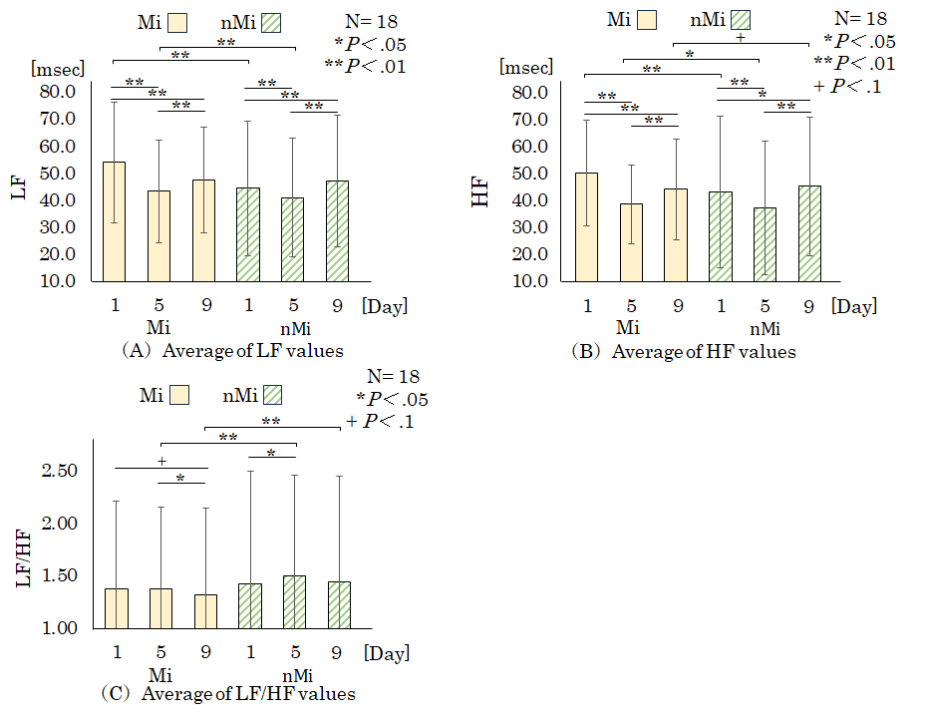
Cardiac potential results. HF: high frequency; LF: low frequency; Mi: mindfulness group; nMi: nonmindfulness group.

The results of the 1-way ANOVA and *t* test for the mean LH value, HF value, and LF/HF ratio are shown in Figures 4A-4C. The horizontal axis shows the date of cardiac potential measurement in the Mi and nMi groups; the vertical axis shows the (A) LH value, (B) HF value, and (C) LF/HF ratio; the error bars in the graph show the SD of each value.

The results of this study showed that the mean LF and HF values of the Mi group were significantly higher on day 1 than on days 5 and 10 (*P*<.01) when comparing each experimental day. In contrast, the LF and HF values of the nMi group were significantly higher on day 1 than on day 5 (*P*<.01) and significantly lower on day 1 than on day 10 (*P*<.05). The LF/HF ratio of the Mi group showed a significant trend on day 1 compared with that on day 10 (*P*<.1) and on day 5 compared with that on day 10 (*P*<.05). The LF/HF ratio in the nMi group was significantly lower on day 1 than on day 5 (*P*<.05).

In a comparison between the groups, LF values on days 1 and 5 were significantly higher than those of the nMi group (*P*<.01); HF values on day 5 were significantly higher than those of the nMi group (*P*<.05); HF values on day 10 showed a significant trend compared to those of the nMi group (*P*<.1); and days 5 and 10 LF/HF ratios were significantly lower than those of the nMi group (*P*<.01).

**Table 3:** Results of multiple comparisons of LH^a^ values, HF^b^ values, and LF/HF ratios.

			Mi^c^		nMi^d^	
			*P* value	Bonferroni	*P* value	Bonferroni
**LF**						
	**Day 1**					
		Day 5	5×10^–9^^e^	2×10^–8^^f^	5×10^–7^^e^	15×10^–6^^f^
		Day 9	5×10^–9^^e^	2×10^–8^^f^	1×10^–4^^e^	4×10^–4^^f^
	**Day 5**					
		Day 9	5×10^–9^^e^	2×10^–8^^f^	5×10^–9^^e^	15×10^–8^^f^
**HF**						
	**Day 1**					
		Day 5	5×10^–9^^e^	2×10^–8^^f^	5×10^–9^^e^	15×10^–8^^f^
		Day 9	5×10^–9^^e^	2×10^–8^^f^	.019	.056
	**Day 5**					
		Day 9	5×10^–9^^e^	2×10^–8^^f^	5×10^–9^^e^	15×10^–8^^f^
**LF/HF ratio**						
	**Day 1**					
		Day 5	.996	2.988	.032^g^	.097
		Day 9	.051	2×10^–1^	.81	2.429
	**Day 5**					
		Day 9	.039^g^	1×10^–1^^h^	.16	.487

^a^LF: low frequency.

^b^HF: high frequency.

^c^Mi: mindfulness group.

^d^nMi: nonmindfulness group.

^e^*P*<.01.

^f^*P*<.003.

^g^*P*<.05.

^h^*P*<.017.

## Discussion

### Sleep Assessment Using a Questionnaire After Mindfulness Practice

This study analyzed the impact of mindful breathing exercises using a tablet device. The questionnaire had a low valid response rate (55.6%). However, of the participants who answered the questionnaire, 100% (n=18) answered that mindfulness meditation affected their sleep quality. In their free-text responses, participants stated that they awoke feeling refreshed and calmer, which made it easier to sleep, and felt that they slept better. Mindfulness is effective against insomnia and mental illness [35]. The results of a subjective evaluation of only those who performed mindful breathing exercises suggested that these exercises not only improved sleep quality but also had a positive effect on mood after waking and falling asleep.

### Sleep Duration

The results of a *t* test comparing sleep duration and each sleep stage (S sleep, PS sleep [shallow], and PS sleep [deep]) showed no significant differences between the groups. This suggests that no significant difference exists in sleep duration between the groups.

Significant differences were found between the mean values of total sleep time and each sleep stage (S sleep, PS sleep [shallow], and PS sleep [deep]) of the groups on each experimental day. When each sleep stage was compared, significant differences were found in the duration of the sleep stages in both groups. Based on the results of this multiple comparison of the 2 groups, it is unclear from the comparison of the total sleep time and time at each sleep stage on each experimental day whether mindful breathing techniques were influential. Future research could assess the impact of mindful breathing techniques by more closely controlling the conditions and situations during sleep.

### Cardiac Potential Measurements

The LF values in the Mi group were significantly lower on days 5 and 10 than on day 1. In evaluating autonomic function using HRV, the LF component reflects sympathetic and parasympathetic activities [31,32]. Mindfulness training decreases the resting LF component [36]. This suggests that suppressing sympathetic activity and activating parasympathetic activity during sleep may be involved during the experiment in the MI group that implemented mindfulness. The LF values in the nMi group were significantly higher on day 5 and lower on day 9 than those on day 1. This suggests that the sympathetic activity during sleep in the nMi group fluctuated during the experiment.

The LF values of the Mi group exhibited a decreasing trend, whereas those of the nMi group fluctuated. This difference may be owing to the difference in the intervention content between the groups. This suggests that activating parasympathetic nerve activity via mindful breathing exercises using the tablet device led to a decrease in LF values.

HF values in the Mi group were significantly higher on day 1 than on days 5 and 9. As the HF component reflects parasympathetic activity [32], parasympathetic activity was dominant on days 5 and 9 compared with the HF values on day 1 in the Mi group. The HF, which indicates parasympathetic activity, increases and affects HRV before and after mindfulness training [36]. These results suggest that mindful breathing exercises activate the parasympathetic system during sleep. The nMi group showed significantly higher HF on day 1 than on day 5 but significantly lower HF on day 1 than on day 9, suggesting fluctuations in parasympathetic activity [32]. This suggests the nMi group showed increased parasympathetic activity from days 1 to 5, followed by suppression on day 9.

The LF/HF ratio in the Mi group was significantly higher on day 9 than on day 1. In contrast, the nMi group showed significantly lower values on day 5 than on day 1. Autonomic control is an interaction rather than the activity of a continuum [37]. Implementing mindfulness leads to an increase in LF power [38]. Implementing mindfulness should have an attention-focusing and relaxation effect [10]. This suggests that continuous mindful breathing practices may calm the mind, alter the LF/HF ratio during sleep, and regulate the balance between sympathetic and parasympathetic nervous system activities.

### Limitations

This study has few limitations. First, the sample size for this study was 9 for each group. This may have led to a decrease in the statistical power of studies assessing autonomic function during sleep. More reliable results can be obtained in the future by expanding the sample size. Second, the HF and LF values for the groups changed compared with those on day 1 but did not remain constant during the experiment. Although some of the implementation methods were standardized, such as the conditions of the participants and operation using the tablet terminal, a more detailed unification of the conditions is required to clarify the characteristics of autonomic nervous activity.

### Conclusions

The results of this study suggest that implementing mindfulness leads to the simultaneous inhibition of parasympathetic activity and an increase in sympathetic activity. Inhibiting sympathetic activity may be reflected in the interaction between autonomic functions. In the Mi group, we observed a suppression of sympathetic activity in LF values and LF/HF ratios during the experiment; in the Mi group, under the conditions of this study, as hypothesized, implementing mindful breathing exercises using the tablet device reduced cardiac potentials, an indicator of autonomic function, has shown a change over time.

These results suggest that in the Mi group, mindful breathing exercises may influence HRV indices during sleep. Future research with larger sample sizes and long-term follow-ups could further validate these findings and inform targeted interventions for sleep-related issues.

## Appendix

Multimedia Appendix 1 CONSORT-eHEALTH checklist (V 1.6.1).

## Abbreviations

HF: high frequency
HRV: heart rate variability
LF: low frequency
Mi: mindfulness group
MBSR: mindfulness-based stress reduction
nMi: nonmindfulness group
PS sleep (deep): deep sleep with parasympathetic nerve dominance
PS sleep (shallow): shallow sleep with parasympathetic nerve dominance
RRI: R-R interval
S sleep: sleep with sympathetic nerve dominance
